# 2D and 3D cultured human umbilical cord-derived mesenchymal stem cell-conditioned medium has a dual effect in type 1 diabetes model in rats: immunomodulation and beta-cell regeneration

**DOI:** 10.1186/s41232-022-00241-7

**Published:** 2022-11-30

**Authors:** Basak Isildar, Serbay Ozkan, Merve Ercin, Selda Gezginci-Oktayoglu, Mahmut Oncul, Meral Koyuturk

**Affiliations:** 1 Department of Histology and Embryology, Cerrahpasa Faculty of Medicine, Istanbul University-Cerrahpasa, Istanbul, Turkey; 2 Department of Biology, Molecular Biology Section, Faculty of Science, Istanbul University, Istanbul, Turkey; 3 Department of Gynecology and Obstetrics, Cerrahpasa Faculty of Medicine, Istanbul University- Cerrahpasa, Istanbul, Turkey

**Keywords:** Mesenchymal stem cell, Human umbilical cord, 3D cell culture, Conditioned medium, Type 1 diabetes

## Abstract

**Background:**

Type 1 diabetes (T1D) is a T-cell-mediated autoimmune disease characterized by the irreversible destruction of insulin-producing β-cells in pancreatic islets. Helper and cytotoxic T-cells and cytokine production, which is impaired by this process, take a synergetic role in β-cell destruction, and hyperglycemia develops due to insulin deficiency in the body. Mesenchymal stem cells (MSCs) appear like an excellent therapeutic tool for autoimmune diseases with pluripotent, regenerative, and immunosuppressive properties. Paracrine factors released from MSCs play a role in immunomodulation by increasing angiogenesis and proliferation and suppressing apoptosis. In this context, the study aims to investigate the therapeutic effects of MSC’s secretomes by conditioned medium (CM) obtained from human umbilical cord-derived MSCs cultured in 2-dimensional (2D) and 3-dimensional (3D) environments in the T1D model.

**Methods:**

First, MSCs were isolated from the human umbilical cord, and the cells were characterized. Then, two different CMs were prepared by culturing MSCs in 2D and 3D environments. The CM contents were analyzed in terms of total protein, IL-4, IL-10, IL-17, and IFN-λ. In vivo studies were performed in Sprague-Dawley-type rats with an autoimmune T1D model, and twelve doses of CM were administered intraperitoneally for 4 weeks within the framework of a particular treatment model. In order to evaluate immunomodulation, the Treg population was determined in lymphocytes isolated from the spleen after sacrification, and IL-4, IL-10, IL-17, and IFN-λ cytokines were analyzed in serum. Finally, β-cell regeneration was evaluated immunohistochemically by labeling Pdx1, Nkx6.1, and insulin markers, which are critical for the formation of β-cells.

**Results:**

Total protein and IL-4 levels were higher in 3D-CM compared to 2D-CM. In vivo results showed that CMs induce the Treg population and regulate cytokine release. When the immunohistochemical results were evaluated together, it was determined that CM application significantly increased the rate of β-cells in the islets. This increase was at the highest level in the 3D-CM applied group.

**Conclusion:**

The dual therapeutic effect of MSC-CM on immunomodulation and homeostasis/regeneration of β-cells in the T1D model has been demonstrated. Furthermore, this effect could be improved by using 3D scaffolds for culturing MSCs while preparing CM.

## Introductıon

Type 1 diabetes (T1D) is an autoimmune disease characterized by the destruction of insulin-producing β-cells in the pancreas, leading to progressive insulin deficiency and consequent hyperglycemia [1]. Hyperglycemia causes complications such as cardiovascular diseases, neuropathy, nephropathy, and retinopathy. Therefore, hyperglycemia should be kept under control to prevent these complications. Today, the most valid treatment method for T1D is insulin injection, which replaces the insulin that the body cannot produce. Exogenous insulin could keep hyperglycemia under control, but it is insufficient to reduce diabetes-related complications because it is not as effective and dynamic as endogenous insulin [2]. It is also ineffective in preventing autoimmunity and regenerating destroyed β-cells [3]. Islet transplantation is another treatment option for T1D, but its applicability is low due to donor scarcity, insufficient cell formation after transplantation, and the risk of immune reaction [4]. It is necessary to develop new treatment strategies that suppress autoimmunity, provide endogenous insulin release, and are applicable to T1D, which affects a large population worldwide.

Mesenchymal stem cells (MSCs) are multipotent precursor cells that show fibroblastic morphology; express cell surface markers such as CD44, CD73, and CD90; and have high proliferation capacities [5]. They can be isolated from many tissues in the body such as the bone marrow, umbilical cord, adipose tissue, and amniotic membrane, and reproduced on a large scale in vitro. Since the umbilical cord is a primitive tissue, cells isolated from it stand out for regenerative treatments with their high proliferation capacity, strong immunomodulation, and immunosuppression abilities. In addition, it is advantageous that cells can be collected without the need for invasive procedures and that the cell source can be reached relatively easily, since the umbilical cord is waste tissue [6]. Recent studies have shown that the therapeutic value of MSCs is related to the secretomes rather than their differentiation ability [7–9]. Appreciations to the secretome, MSCs have a therapeutic effect by playing a role in mechanisms such as tissue repair, immunomodulation, and immunosuppression. It has been revealed that the secretome consists of cytokines such as IL-4; IL-6; IL-10; growth factors such as TGF-β1, HGF, and VEGF; and immunomodulatory molecules such as HLA-G, PGE2, and chemokines [10]. On the other hand, insoluble factors are membranous particles of various sizes and contents that enable the transport of microRNAs and proteins that play a role in cell-cell interaction [11, 12]. MSCs secrete the secretomes into the medium in which they are grown, and it is defined as the conditioned medium. The conditioned medium has therapeutic value and acts through autocrine/paracrine mechanisms in pathophysiological processes [13]. When MSCs are expected to exert a therapeutic effect through the factors they secrete, it is advantageous to use the conditioned medium instead of the cells themselves. In this way, it is possible to develop a more controlled and directive treatment option, and at the same time, risks such as the cells going and settling in unwanted places, differentiation into unwanted cell types, and the possibility of forming tumors are not taken [8, 9]. In addition, since pro-inflammatory cytokines formed in the organism due to the target disease may adversely affect the biological properties of transplanted MSCs, it seems more advantageous to use cell products instead of the direct use of cells [14].

Increasing the activity of MSCs with various external stimuli and enabling them to secrete at a higher level per unit area improves the therapeutic effectiveness of the conditioned medium. These methods can be classified as hypoxic conditioning, using chemical agents, 3-dimensional (3D) scaffolds, and inflammatory cytokines. 3D culturing of MSCs in vitro increases the paracrine secretion capacity of these cells [15]. Because cell culture analyses in a 2D environment cannot mimic the complex biological functions of cells, such as extracellular matrix interactions, migration, transcriptional regulation, and receptor expression in their physiological environment, translation of the outcomes to the clinic is insufficient. 3D culture medium handles this challenge and serves as a better model that closely represents in vivo physiological conditions [16]. Studies have shown changes in the gene and protein expression profiles of cells cultured in 3D and the amounts of paracrine factors they secrete. Growing cells by following a particular topography on scaffolds that mimic the extracellular matrix may change the secretome profiles of MSCs and intensify the conditioned medium content [17]. Alvetex® 3D scaffolds used to prepare 3D conditioned medium in this study are highly porous, cross-linked polystyrene material that does not degrade during use, and it provides a suitable 3D environment for cells to perform their growth, migration, and differentiation functions [8]. Since the material of the 2D culture dishes is polystyrene, the material of the scaffolds is also chosen so that the material-cell interaction is not different.

In the present study, we aimed to investigate the effects of CM obtained by 2D and 3D culturing of the human umbilical cord-derived MSCs and reveal the therapeutic effects of two different CMs on the autoimmune T1D model in the context of immunomodulation and β-cell regeneration.

## Materials and methods

### Ethics statement

This study was approved by the Clinical Trials Ethics Committee of Cerrahpasa Faculty of Medicine (Approval number: 83045809-604.01.02), and the umbilical cord was collected with written consent from the patient. In order to keep the risk of contamination low, the patient who had a cesarean section was preferred. The animal experiments were conducted following Turkey’s Regulation of Animal Research Ethics. The ethical approval required for animal experiments was granted by the Bezmialem Vakıf University Local Ethics Committee (Ethics Committee No: 2019/38), and forty-eight male Sprague-Dawley rats were used.

### Isolation of UC-MSC

MSCs were isolated from the umbilical cord by the tissue explant method. Leibovitz L-15 Medium (PanBiotech, Germany) with 1% (v/v) of amphotericin B (Sigma, USA) was used to transport the umbilical cord. The cord tissue was washed with phosphate-buffered saline (PBS) to eliminate the blood clots, and two arteries and one vein in the cord were carefully removed. The tissue explant method was briefly applied as follows. First, the cord tissue was divided into 1–2-mm^3^ pieces. Then, the pieces were placed onto sterile and plastic Petri dishes and incubated in a culture medium [Dulbecco’s modified Eagle’s medium (DMEM/F12:1/1: Sigma, USA)] supplemented with 10% (v/v) of fetal bovine serum (FBS: Hyclone, UK), 1% (v/v) of penicillin/streptomycin (Hyclone, UK), and amphotericin B (2.5 μg/ml) for 3 h at 37 °C in a humidified atmosphere, with 5% CO_2_. Then, 6 ml of medium was added to the tissue pieces. The medium in the Petri dishes was carefully renewed three times a week. The areas around the tissue pieces were examined with an inverted microscope (Olympus IX71), and the isolation was followed. After the cells reached sufficient confluency, the tissue pieces were removed from the Petri dish, and when the confluency reached 80–90%, the cells were passaged and were obtained from the first passage [6].

### Characterization of UC-MSC

#### Immunophenotypic characterization

When the isolated cells reached the third passage in the culture conditions, their cell surface antigen expressions were evaluated by flow cytometric analysis for characterization. For this purpose, MSC-specific markers CD44-PE and CD90-APC were analyzed as positive markers, and endothelial CD34-FITC and hematopoietic CD45-APC-Cy were used as negative markers for MSCs (BD the Biosciences, USA) [18]. Isotype antibodies determined for each antibody were used as negative control (Biolegend, San Diego; APC mouse IgG1, κ isotype control, APC/Cyanine7 mouse IgG1, κ isotype control, PE mouse IgG1, κ isotype control, FITC mouse IgG1, κ isotype control). Briefly, the cells were first collected by trypsinization and pelleted by centrifugation at 1500 rpm for 5 min. The number of viable cells was determined by staining the cells with trypan blue and counting. 10^6^ of cells were pipetted through a 70-μm filter, and the cells were separated from each other for flow cytometry analysis. After washing twice in staining buffer (2% FBS in PBS), primary and isotype antibodies were added to the mixtures and incubated in the dark for 30 min. After incubation, the cells were washed two more times with staining buffer and then analyzed by BD FACS Callibur [6, 18]. This analysis was repeated three times, and the results are given as mean + SD.

#### MSC differentiation into adipocyte and osteoblast lineages

Differentiation was performed following the instructions of commercially purchased adipogenic and osteogenic differentiation medium. For adipogenic differentiation, the cells at the third passage were seeded in a 12-well plate at 1.6 × 10^6^ per well. After the cells reached 70% confluency in standard culture conditions, the medium was replaced with the adipogenic differentiation medium (Sigma: 417D-250). During the differentiation period, the differentiation medium was refreshed every 3 days. At the end of the 21st day, differentiation-induced cells were fixed with 4% paraformaldehyde for 20 min after washing with PBS. After fixation, washing with PBS was repeated, and cells were incubated with Oil Red O for 20 min to show the cell’s lipid content. Following staining, cells were washed with PBS until the background became translucent.

For osteogenic differentiation, cells in the third passage were seeded in a 12-well plate at 8× 10^5^ per well. After the cells reached 70% confluency in the normal culture medium, the medium was replaced with the osteogenic differentiation medium (Sigma: 811D-250). The osteogenic differentiation medium was refreshed every 3 days during the differentiation period. At the end of the 21st day, Alizarin Red staining was performed to show extracellular calcium accumulation due to the differentiation process. For this purpose, the cells were washed with PBS and fixed with 4% paraformaldehyde for 20 min. After repeating the washing step, the cells were incubated with Alizarin Red for 45 min in the dark and at room temperature and then washed with distilled water until the background became translucent. The cells undergoing adipogenic and osteogenic differentiation were examined and photographed with the BioTek LionHeart FX microscope.

### Preparation of 2D and 3D conditioned medium

Conditioned medium prepared in two different ways were used to treat T1D. The first was a 2-dimensional (2D) traditional culture environment (2D-CM), while the other was a 3-dimensional (3D) Alvetex® scaffold (3D-CM). Briefly, the conditioned medium preparation steps are as follows. First, the wells of the Alvetex® culture dishes were washed with 70% ethanol and then with the culture medium twice. When the cells were in the third passage, they were seeded into 12-well culture dishes at 2 × 10^5^ cells in 3 ml of medium. In both conditions, cells were first cultured in culture medium [fetal bovine serum (FBS), DMEM F12 containing 1% penicillin-streptomycin/amphotericin B], in an incubator containing 5% CO_2_ at 37 °C. The cells in the 2D culture medium were followed to reach 80% density, and then the medium of both 2D and 3D media was replaced with a serum-free medium. The resulting medium was collected cell-free after the cells were cultured in this medium for 48 h. The remaining cells were trypsinized, and the viability was evaluated by staining with trypan blue. The medium obtained from the wells in which the viability was preserved above 80% was called the conditioned medium. Following centrifuging and clearing the cell debris, the conditioned medium was passed through a 0.22-μm filter and stored at − 80 °C until used [19, 20].

### Content analysis 2D and 3D conditioned medium

The total protein content of the conditioned medium obtained from MSCs in 2D and 3D cultures was measured by the bicinchoninic acid (BCA) method, a colorimetric method. The procedure was performed with the directions in the commercially purchased protein quantification kit (KTD3001, Abbkine Co., USA), the absorbances were measured with a microplate reader (Allsheng AMR-100 Microplate Reader AS-16050-00), and the protein amount was calculated as μg/ml. For the cytokine analysis in the conditioned medium, commercially purchased “enzyme-linked immunosorbent assay” (ELISA) kits were used, and the levels of IFN-γ (EK0373, Boster, Wuhan, China), IL 17 (EK0430, Boster, Wuhan, China), IL-4 (EK0404, Boster, Wuhan, China), and IL-10 (EK0416, Boster, Wuhan, China) were detected. The manufacturer’s instructions were followed during the analyses, and the measurements were made with a 450-nm microplate reader (Allsheng AMR-100 Microplate Reader AS-16050-00). Each sample was performed in duplicates to reduce the error. The concentrations of the samples were calculated as pg/ml.

### Experimental design and induction of autoimmune T1D model

In order to examine the in vivo effects of the 2D and 3D CMs on the T1D model, 10–12-week-old Sprague-Dawley-type male rats with a body weight of 350–450 g were used. They were housed at constant room temperature for 12-h light and dark cycles and fed standard laboratory chow and water ad libitum. The animals were randomly divided into six groups first. Then, to establish an autoimmune type 1 diabetes model, 20 mg/kg streptozotocin (STZ) (Sigma-Aldrich) freshly dissolved in citrate buffer (0.1 M, pH 4.5) was administered intraperitoneally once a day for five consecutive days [21]. Only citrate buffer was applied to the control group rats for the same period. During this process, blood glucose was monitored with a glucometer in the blood taken from the tail vein of the rats, and rats with blood glucose higher than 250 mg/dl were considered diabetic [22]. The rats’ blood glucose values and weights were monitored weekly until the experiment was terminated. The experimental groups were assembled as given in Table 1.

**Table 1:** The experimental groups

Control group (C):Rat group treated with citrate buffer	8	Diabetes group (D):Rat group treated with STZ in citrate buffer	8
Control + conditioned medium with the 2D culture of MSCs (C+2D-CM)	8	Diabetes + conditioned medium with the 2D culture of MSCs (D+2D-CM)	8
Control + conditioned medium with the 3D culture of MSCs (C+3D-CM)	8	Diabetes + conditioned medium with the 3D culture of MSCs (D+3D-CM)	8

### Treatment procedure

**Fig. 1:**
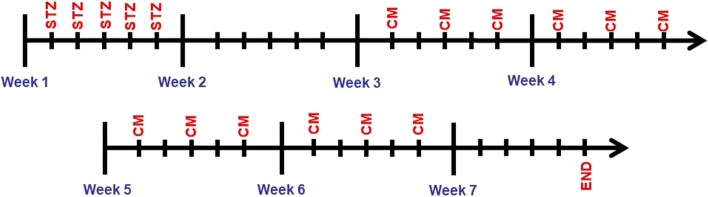
Timeline of disease modeling and treatment process

As mentioned in Table 1, the first diabetic group did not receive any intervention during the treatment period. In the third week of the experimental procedure, 2D-CM was applied to the second diabetic group and 3D-CM to the third diabetic group, three times a week, 1 ml intraperitoneally, and this application was continued for 4 weeks. Similarly, no treatment was applied to the first control group during the treatment period. 2D-CM was applied to the second control group, and 3D-CM to the third control group was applied intraperitoneally simultaneously with the experimental groups, and this application was continued for 4 weeks. The rats were sacrificed under ketamine/xylazine 1 week after the last CM administration. The animals’ blood glucose levels and weights were monitored throughout the experiment. The time flow regarding the creation of the disease model and the treatment process is given in Fig. 1.

### Treg population analysis

Treg cell population analysis was performed on the mononuclear cells isolated from the spleen, and the following protocol was applied [23]. Briefly, 1/3 of the spleen tissue, which was taken into RPMI medium (21875034, Thermo Fisher), was used, transferred to a Petri dish, and cut into coarse pieces. In order to isolate the mononuclear cells, spleen tissue was mashed properly between two slides, and the slides were washed with Hanks’ Balanced Salt Solution (HBSS). Thus, a cell solution was created in the Petri dish and collected in a Falcon tube. The solution’s volume was completed to 25 ml by adding HBSS. It was then centrifuged at + 4 °C at 2000 RPM for 10 min. In order to remove the erythrocytes, the pellet was dissolved with 5 ml of 1× erythrocyte lysis buffer (420301, Biolegend) and incubated for 3 min at room temperature. After incubation, the solution was made up to 40 ml by cold PBS. It was centrifuged for 10 min at 2000 RPM. In the last step, the supernatant was discarded, and the cell pellet was dissolved in 30 ml of medium. The solution was passed through a 70-μm cell filter, and the viability was controlled with trypan blue analysis. Then, flow cytometric analysis was performed on the cells.

The cells were analyzed for CD4 (201509, Biolegend), CD25 (202103, Biolegend), and FoxP3 (320008, Biolegend) by using specific primary antibodies with flow cytometry to determine the Treg ratio in the isolated cell population. Briefly, approximately 2 × 10^6^ cells were washed with 2 ml of PBS, then the cells were centrifuged at 3000 rpm for 5 min, and the pellet was dissolved with 100 μl of staining solution. CD4 and CD25 antibodies were added to the solution and incubated for 45 min in the dark at + 4 °C. After incubation, 500 μl of staining solution was added to the tubes and centrifuged. The pellet was dissolved by discarding the supernatant, and this step was repeated. Subsequently, the pellet was dissolved with 500 μl of permeabilization and fixation solution (424401, Biolegend) and incubated overnight at + 4 °C. After incubation, the tubes were centrifuged at + 4 °C and 3000 rpm for 5 min. The cell pellet was thawed with permeabilization wash buffer (424401, Biolegend). After 15 min of incubation, it was centrifuged again. Finally, cells were labeled with FoxP3 antibody and incubated for 1 h at + 4 °C. Then, permeabilization wash buffer was added to the tubes, and the tubes were centrifuged at + 4 °C, 3000 rpm for 5 min. The final cell pellet was dissolved in 500 μl of staining solution, and analysis was performed with the BD FACS Callibur system [23]. The results are given as mean + SD.

### ELISA analysis for C-peptide, insulin, IL-4, IL,10, IL-17, and IFN-γ

Cardiac blood was collected from animals at week 7 for cytokine analyses. Serum levels of C-peptide Cloudclone (CEA447Ra, Uscn Life Science Inc. Wuhan, China), insulin (CEA448Ra, Uscn Life Science Inc. Wuhan, China), Th1-related IFN-γ (SEA049Ra, Uscn Life Science Inc. Wuhan, China), Th17-related IL-17 (SEA063Ra, Uscn Life Science Inc. Wuhan, China) as pro-inflammatory, and Th2-related IL-4 (SEA063Ra, Uscn Life Science Inc. Wuhan, China) and Treg-related IL-10 (SEA056Ra, Uscn Life Science Inc. Wuhan, China) cytokines as anti-inflammatory were evaluated by ELISA analysis. The manufacturer’s instructions were followed during the analyses, and the measurements were made with a 450-nm microplate reader (Allsheng AMR-100 Microplate Reader AS-16050-00). With the measured absorbance values, the concentrations of the samples were calculated as pg/ml.

### Histological analysis

The pancreatic tissues were fixed with a 10% neutral buffered formalin solution, dehydrated with ethanol, then embedded in paraffin for light microscopic analysis. Four-micrometer μm sections were taken from the paraffin blocks and stained with hematoxylin-eosin (H&E) for histological examinations. Briefly, hematoxylin was applied to the sections for 20 min, bruised in tap water for 15 min, then stained with eosin and closed by passing through toluene with a rising alcohol series. As a result of this staining, the nuclei were stained purple and cytoplasm pink. The H&E-stained sections were examined to evaluate the islet morphology with the Olympus BX61 light microscope [24]. In addition, the area of 20 islets per group was measured with the ImageJ program, and the average size of the islets was calculated.

### Immunohistochemical analysis

The pancreas sections were labeled immunohistochemically for Pdx1 and Nkx6.1 markers, which are critical for the formation of β-cells, and the β-cell ratio was evaluated. To determine the insulin expression of β-cells, the sections were labeled with the anti-insulin antibody. Immunohistochemistry applications were performed as follows [25]. First, the sections were deparaffinized in toluene and then rehydrated in graded series of ethanol. The slides were kept in Tris/EDTA (pH = 9.0) for Nkx6.1 and insulin and in citrate buffer (pH = 6.0) for Pdx1 10 × 2 min to perform antigen retrieval step in the microwave oven. After washing in Tris buffer saline (TBS), endogenous peroxidase activity was inhibited by applying 3% H_2_O_2_ (Merck, Darmstadt, Germany) to the sections for 10 min. TBS wash was repeated, then the sections were kept in the blocking solution (SHP125; ScyTek, West Logan, UT) for 10 min for Nkx6.1 and insulin antibodies and 1 h for Pdx1 antibody to prevent non-specific antibody binding. After the blocking step, the sections were incubated overnight at + 4 °C at the indicated dilutions with anti-Pdx1 (1/500, ab47267, Abcam, USA), anti-Nkx6.1 (1/200, ab221544, Abcam, USA), and anti-insulin (1/500, ab181547 Abcam, USA) antibodies. Following incubation, the sections were washed with TBS, kept in the biotinylated secondary antibody solution (SHP125; ScyTek, West Logan, Utah) for 10 min, and washed again. Then, streptavidin peroxidase solution (SHP125; ScyTek, West Logan, UT) was applied to the sections for 10 min. The reaction was made visible with the 3-amino-9-ethyl carbazole (AEC) (ACJ500; ScyTek, West Logan, UT). The samples have been counterstained with Mayer’s hematoxylin (Thermo Fisher, Waltham, MA, USA) and examined with light microscopy (Olympus BX61). During the evaluation, 7–10 islets per animal for Nkx6.1 and 5 islets per animal for Pdx1 were examined. The cells were counted, and the number of positive cells in an islet was calculated as a percentage. In order to evaluate the insulin antibody, an average of 7 islets were examined for each animal, and the percentage of the area showing insulin positivity in an islet in the total islet area was calculated with the ImageJ program.

### Statistical analysis

The SPSS version 21.0 statistical program was used for statistical analysis, and data were expressed as mean ± standard error of the mean. The normality of the data was evaluated with the Shapiro-Wilk test. The mean of more than two groups with normal distribution was compared with parametric one-way analysis of variance (one-way ANOVA), and the significance between the groups was analyzed using the post hoc Tukey HSD test. In cases where data were not normally distributed, the significance between the groups was evaluated using the non-parametric Kruskal-Wallis test. A value of *p* < 0.05 was considered statistically significant.

## Results

### Isolation, immunophenotypic characterization, and differentiation analyses of MSCs

**Fig. 2:**
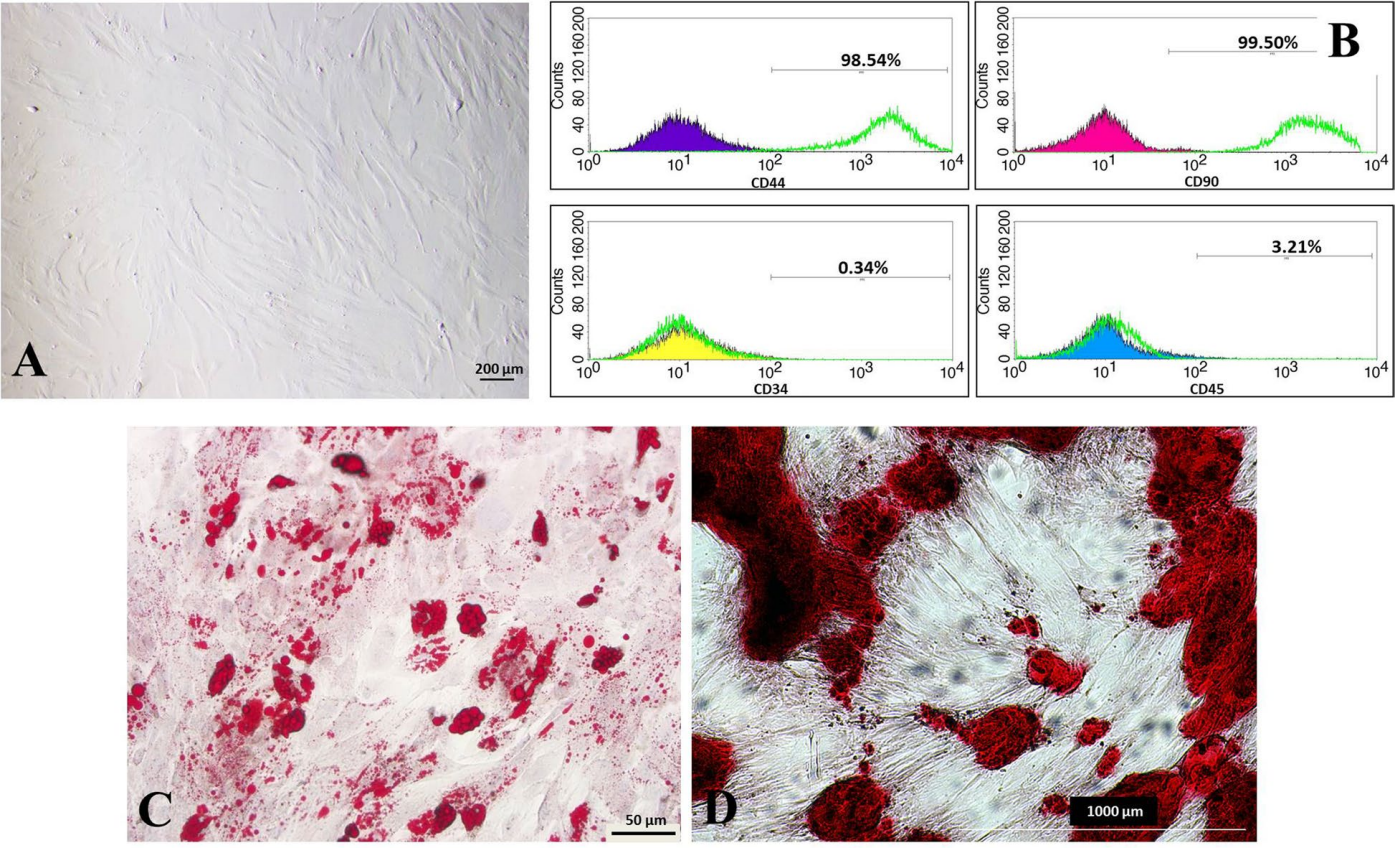
Characterization results of mesenchymal stem cells. **A** Mesenchymal stem cells with spindle- and star-shaped morphology. **B** Immunophenotypic characterization of umbilical cord-derived MSCs. **C** Adipogenic differentiation of MSCs at the end of the 3rd week, showing lipid droplets, Oil Red O. **D** Osteogenic differentiation of MSCs at the end of the 3rd week, showing calcium mineralization, Alizarin Red

MSCs isolated from the human umbilical cord were plastic adherent and proliferative cells showing characteristically spindle- and star-shaped morphology (Fig. 2A). Flow cytometric analysis showed that they were positive for MSC markers CD44 (98.15 ± 0.86%) and CD90 (99.50 ± 0.22%) while negative for CD34 (0.34 ± 0.02%) and CD45 (3.21 ± 2.73%) (Fig. 2B). The differentiation experiments demonstrated that the cells have adipogenic and osteogenic differentiation potential (Fig. 2C, D).

### Content analysis 2D and 3D conditioned medium

The protein content of the 2D-CM and 3D-CM was measured by the BCA method. The analysis revealed that the protein concentration in 2D-CM was 47,931 μg/ml, and in 3D-CM, it was 64,310 μg/ml. When CM contents were evaluated by ELISA analysis in terms of cytokine levels, IL-4, an anti-inflammatory cytokine, was 17.11 pg/ml in 2D-CM and 23.25 pg/ml in 3D-SM. For IL-10, an anti-inflammatory cytokine, and IL-17 and IFN-γ, pro-inflammatory cytokines, no measurable protein level could be detected in 2D-CM and 3D-CM.

### Body weight change and blood glucose analysis in experimental animals

**Fig. 3:**
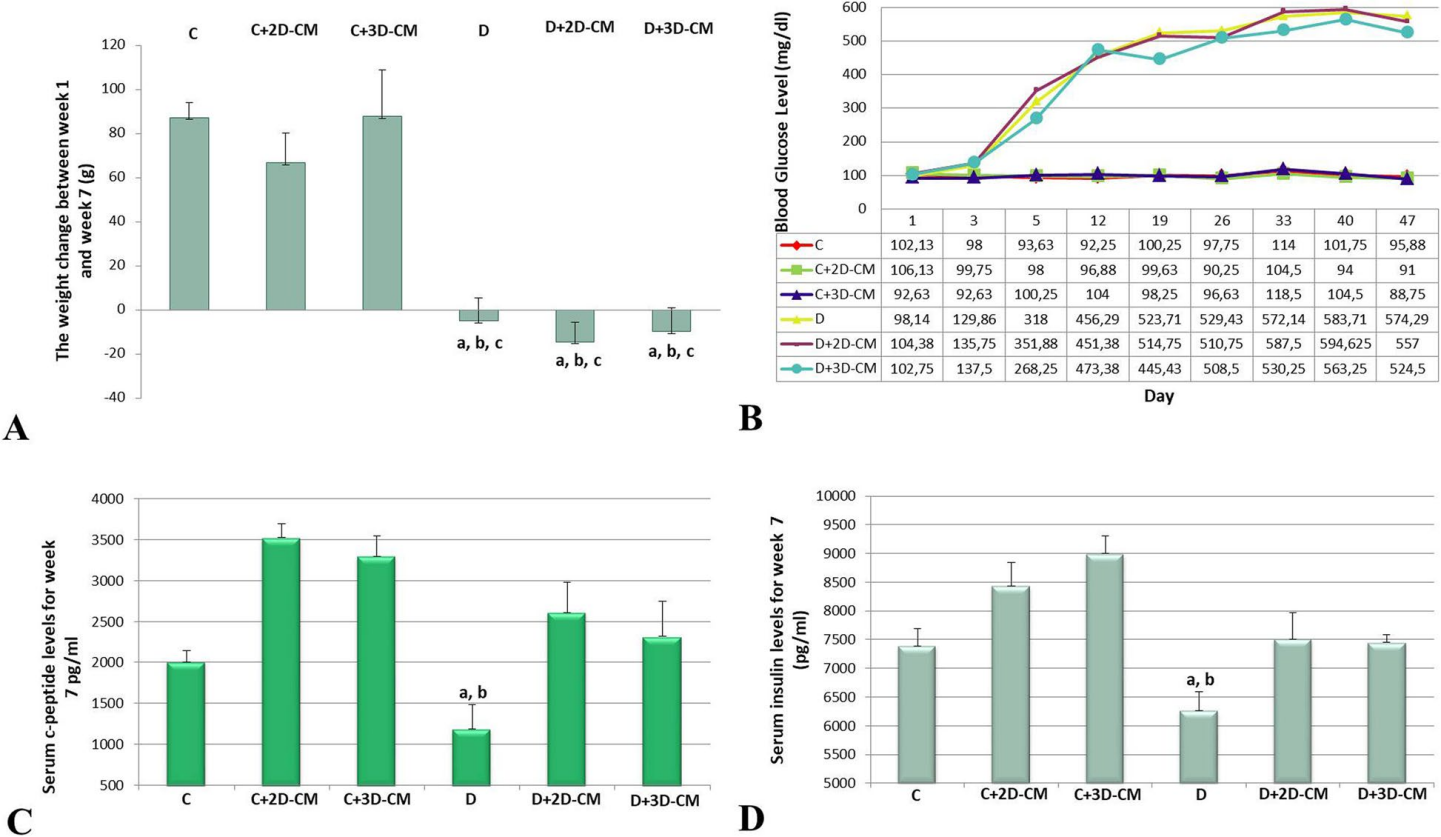
Body weight, blood glucose, serum C-peptide, and serum insulin analysis. **A** Weight change of the animals between 1st week and 7th week (g). ^a^*p* < 0.001 versus the C group, ^b^*p* < 0.01 versus the C+2D-CM group, ^c^*p* < 0.001 versus the C+3D-CM group. Data are presented as mean ± SEM (*n* = 8). **B** Mean blood glucose levels of the animals during the experiment period (*n* = 8). **C** Serum C-peptide levels of the groups, ^a^*p* < 0.01 versus the C+2D-CM group, ^b^*p* < 0.05 versus the C+3D-CM group. **D** Serum insulin levels of the groups, ^a^*p* < 0.05 versus the C+2D-CM group, ^b^*p* < 0.01 versus the C+3D-CM group. Data are presented as mean ± SEM (*n* = 5)

The body weights of the experimental animals followed for 7 weeks were examined, and it was detected that there was no sudden weight loss due to STZ application. However, when the weight changes calculated over the values at the beginning and end of the experimental procedure were compared, a statistically significant difference has seen among the groups. The D, D+2D-CM, and D+3D-CM groups showed significantly higher weight loss than the C groups (C, C+2D-CM, C+3D-CM). The data are presented in Fig. 3A. When blood glucose levels were examined, it was observed that the animals’ blood glucose levels increased gradually during the STZ administration. In the second week, blood glucose levels were measured above 250 mg/dl in all animals treated with STZ. Thus, the T1D model was established, and the animals maintained their diabetic status until the end of the experiment. No significant difference was seen among the groups at the beginning of the experimental period (day 1). However, the data at the end of the experimental period (47th day) were analyzed, and it was noticed that there was a significant difference among the groups (*p* < 0.001). The D, D+2D-CM, and D+3D-CM groups showed significantly higher blood glucose levels than the C groups (C, C+2D-CM, C+3D-CM). The average data of the groups regarding weekly blood glucose levels and the data graph are given in Fig. 3B.

### Serum C-peptide and insulin analysis

C-peptide levels were used to confirm diabetes classification. The low C-peptide level associated with high blood glucose indicated that the model was compatible with T1D. Serum C-peptide and insulin analysis were performed by the ELISA method in the blood taken from non-fasting animals, just before sacrification at week 7. C-peptide levels showed that there was a significant difference between the groups (*p* < 0.01), and this difference was caused by the C+2D-CM and D groups (*p* < 0.05) and the C+3D-CM and D groups (*p* < 0.01). Consistent with C-peptide results, insulin levels revealed a significant difference between the groups (*p* < 0.01), and this significance was evident for the C+2D-CM and D groups and the C+3D-CM and D groups. The results are presented in Fig. 3C, D.

### Treg population analysis

**Fig. 4:**
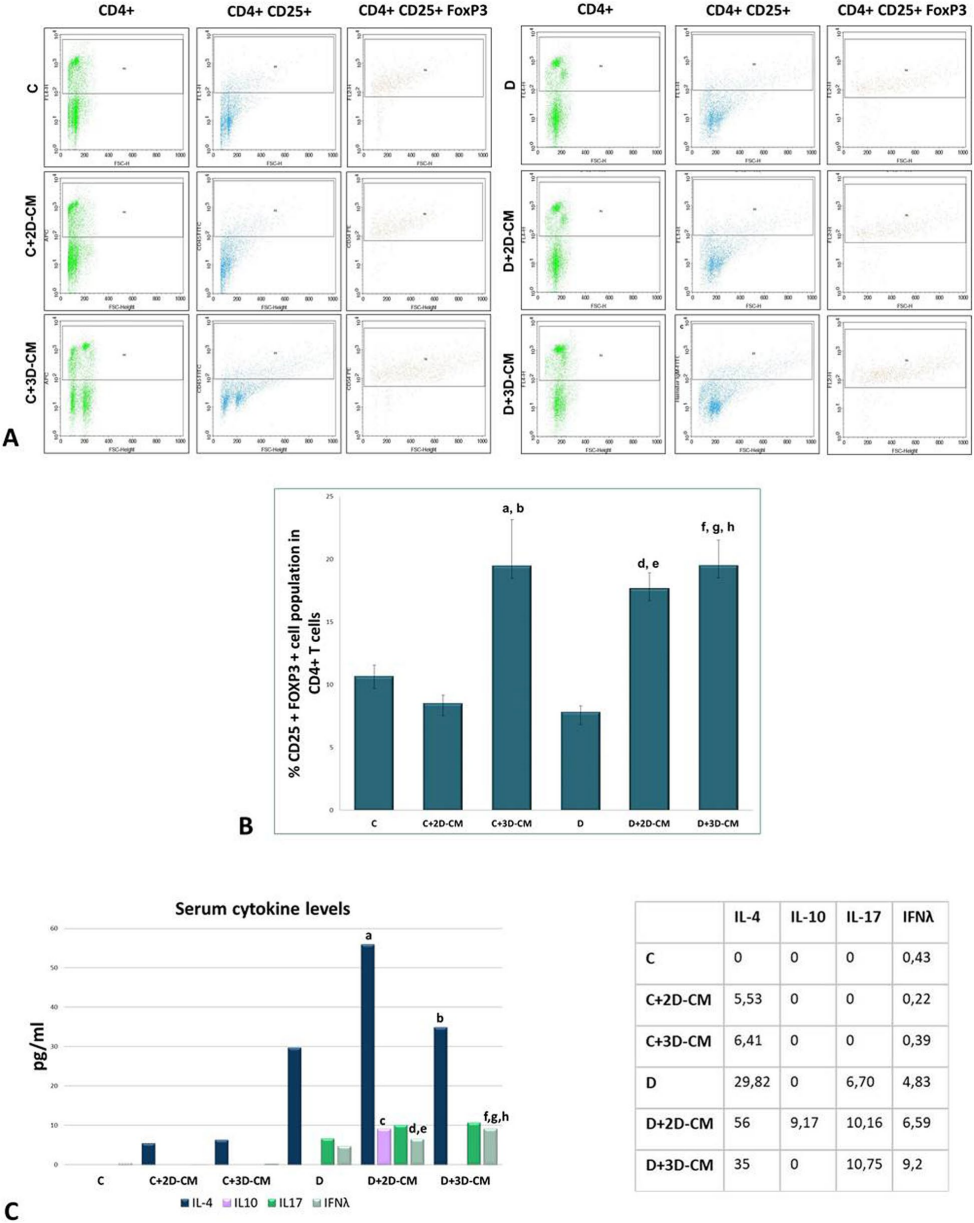
Treg and cytokine analysis. **A** Representative dot blots showing the CD4+ cell population in lymphocytes isolated from the spleen. **B** Representative dot blots showing the CD25+ cell population within the CD4+ cells. **C** Representative dot blots showing the FoxP3 cell population in the CD4+CD25+ cells. **D** Percentage of CD25+ Foxp3+Treg in the CD4+ lymphocytes isolated from the spleen. ^a^*p* < 0.05 versus the C+2D-CM group, ^b^*p* < 0.05 versus the C+2D-CM group, ^c^*p* < 0.01 versus the C+3D-CM group, ^d^*p* < 0.05 versus the C+2D-CM group, ^e^*p* < 0.01 versus the D group, ^f^*p* < 0.05 versus the C group, ^g^*p* < 0.01 by the C+2D-CM group, ^h^*p* < 0.01 versus the D group. E- Cytokine levels of the groups. ^a^*p*<0.01 versus the C group (IL-4), ^b^*p* < 0.05 versus the C group (IL-4), ^c^*p* < 0.05 versus all groups (IL-10), ^d^*p* < 0.05 versus the C group (IFNγ), ^e^*p* < 0.05 versus the C+2D-CM group (IFNγ), ^f^*p* < 0.01 versus the C group (IFNγ), ^g^*p* < 0.001 versus the C+2D-CM group (IFNγ), ^h^*p* < 0.05 by the C+3D-CM group (IFNγ). Data are presented as mean ± SEM (*n* = 8)

To evaluate the immunomodulatory properties of 2D and 3D conditioned medium, the percentage of Treg, which is known to play an essential role in preventing autoimmune reaction by regulating T-cell proliferation and cytokine production, was determined by flow cytometric analysis in the mononuclear cells isolated from the spleen. This analysis was performed by selecting CD25 and Foxp3-positive cells within CD4-positive T-cells. The results obtained in spleen mononuclear cells showed that the Treg population was 10.7 ± 0.86% in the C group, 8.54 ± 0.62% in the C+2D-CM group, and 19.49 ± 3.65% in the C+3D-CM group. The Treg population percentage was 7.83 ± 0.49% in the D group, 17.67 ± 1.23% in the D+2D-CM group, and 19.51 ± 2.01% in the D+3D-CM group. Considering these results, it was observed that the percentage of Treg in the D group decreased relatively compared to the C groups. In the D+2D-CM and D+3D-CM groups, the percentage of Treg showed a significant increase compared to the D group. In addition, it was remarkable that the increase in Treg in the C+3D-CM group with healthy rats treated with 3D conditioned medium was statistically significant compared to the C and C+2D-CM groups. The flow cytometric gating strategy is given in Fig. 4A, and the graph created over the averages of these data is given in Fig. 4B.

### ELISA analysis for IL-4, IL,10, IL-17, and IFN-γ

Blood samples were collected from the animals to investigate the immunomodulatory properties of CMs. IL-4, IL-10, IL-17, and IFN-λ levels were determined by ELISA analysis in the serum obtained from the blood collected before the sacrification of the animals. When the IL-4 results were analyzed, it was seen that there was a significant difference between the groups (*p* < 0.001). There was an increase in IL-4 levels in the diabetes groups compared to the control groups, and the increase in the D+2D-CM (*p* < 0.01) and D+3D-CM (*p* < 0.05) groups were statistically significant compared to the C group. When it comes to IL-10 level analysis, it was seen that there was no measurable level of IL-10 in the groups except the D+2D-CM group. The IL-10 level in the D+2D-CM group was determined as 9173 pg/ml on average, and this increase was found to be statistically significant (*p* < 0.01). While IL-17 levels, one of the pro-inflammatory cytokines, could not be detected in the C groups, they increased in the D groups. However, this increase was not statistically significant. Finally, IFN-λ levels were evaluated, and it was observed that there was a significant difference between the groups (*p* < 0.001). Cytokine level graphs are given in Fig. 4C.

### Histological analysis

**Fig. 5:**
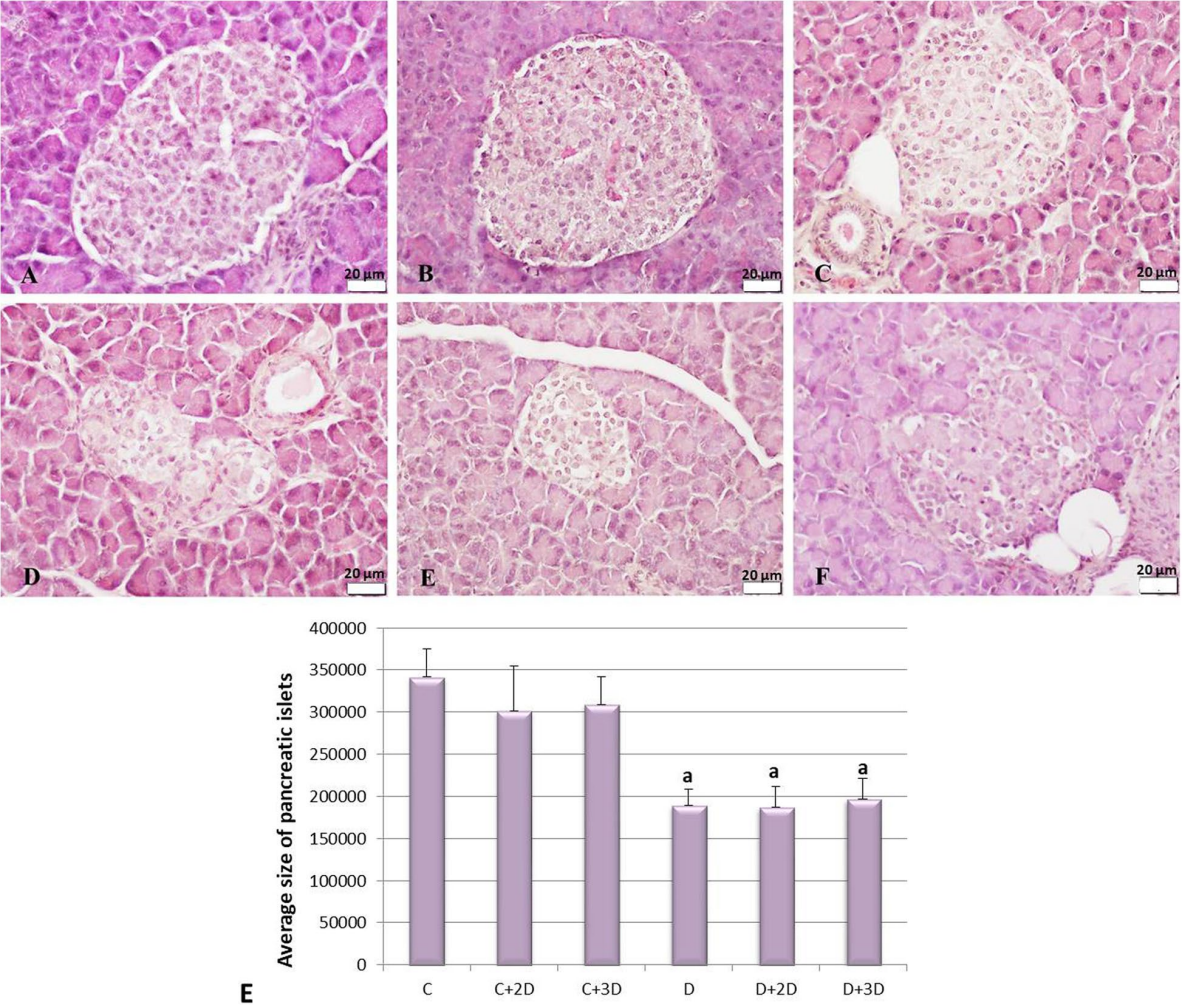
Histological examinations. **A** C group. **B** C+2D-CM group. **C** C+3D-CM group. **D** D group. **E** D+2D-CM group. **F** D+3D-CM group. H&E, × 40. **G** Average size of pancreatic islets (pixels), ^a^*p* < 0.05 versus the C group

Pancreatic sections were stained with H&E to evaluate islet morphology. As a result of the evaluations, islets with irregular contours and smaller sizes were observed in the diabetes groups compared to the control groups. The decrease in islet sizes was statistically significant (*p* < 0.001). Pancreatic islet micrographs stained with H&E are given in Fig. 5A–F, and the average size of the islets is in Fig. 5G.

### Immunohistochemical analysis

#### Nkx6.1

**Fig. 6:**
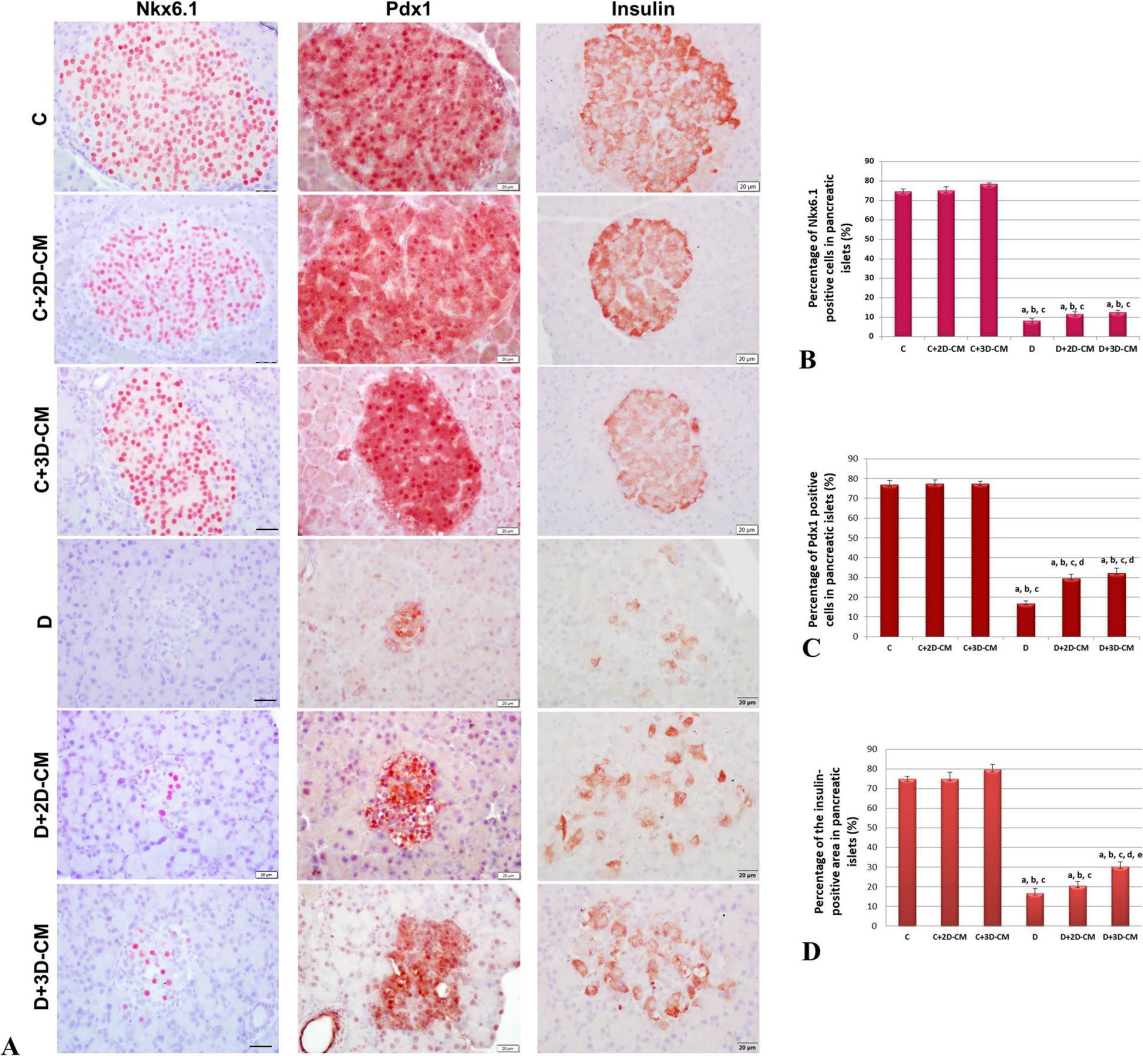
Immunohistochemical analyzes. **A** Immunohistochemical analyzes showing numbers of the Nkx6.1, Pdx1-positive, and insulin-positive areas in the pancreatic islets, × 40. **B** Percentage of the Nkx6.1-positive cells in the islets. ^a^*p* < 0.001 versus the C group, ^b^*p* < 0.001 versus the C+2D-CM group, ^c^*p* < 0.001 according to the C+3D group. **C** Percentage of the Pdx1-positive cells in the islets. ^a^*p* < 0.001 versus the C group, ^b^*p* < 0.001 versus the C+2D-CM group, ^c^*p* < 0.001 versus the C+3D-CM group, ^d^*p* < 0.001 according to the D group. **D** Percentage of insulin positivity in the islets. ^a^*p* < 0.001versus the C group, ^b^*p* < 0.001 versus the C+2D-CM group, ^c^*p* < 0.001 versus the C+3D-CM group, ^d^*p* < 0.01 versus the D group, ^e^*p* < 0.05 versus the D+2D-CM group. Scale bar: 20 ul

**Fig. 7:**
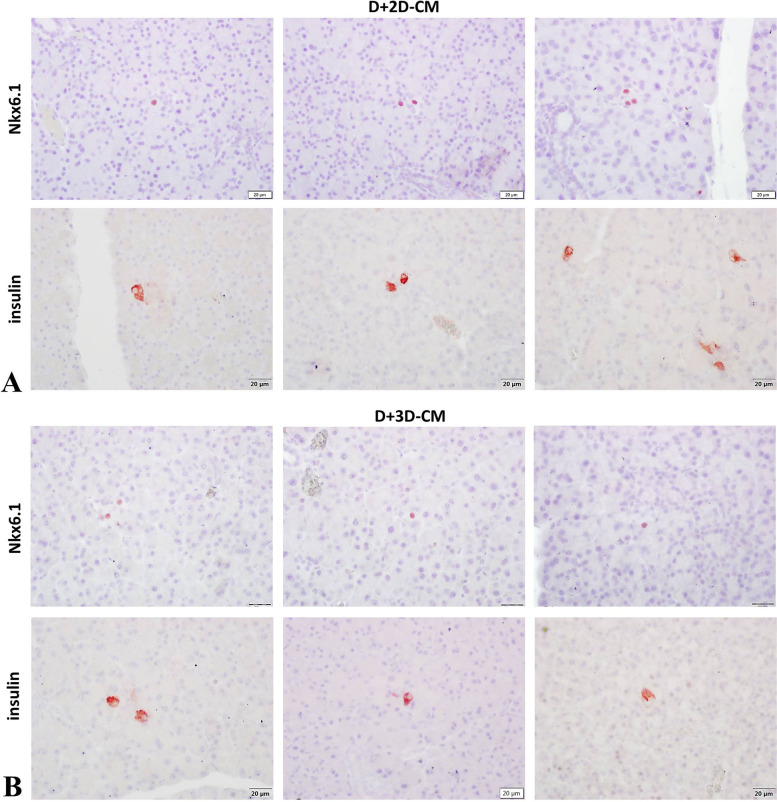
Nkx6.1 and insulin-positive cells in the exocrine pancreas. **A** Micrographs showing Nkx6.1 and insulin-positive cells in the exocrine pancreas in the D+2D-CM group. **B** Micrographs showing Nkx6.1 and insulin-positive cells in the exocrine pancreas in the D+3D-CM group. Scale bar: 20 um

To evaluate the effects of 2D-CM and 3D-CMs on β-cell homeostasis and regeneration on the T1D model, Nkx6.1, a β-cell marker, was labeled immunohistochemically in the pancreatic sections (Fig. 6A). Then, 7–10 sections were examined for each animal. Nkx6.1-positive cells with nuclear staining were counted in the sections, and the percentage of these cells in the islets was calculated (Fig. 6B). Accordingly, the rate of positive cells in the islets was determined as 74.44% in the C group, 74.95% in the C+2D-CM group, and 78.33% in the C+3D-CM group. In the D group, this positivity was detected at a rate of 8.14% with weakened expression intensity. In the D+2D-CM and D+3D-CM treatment groups, Nkx6.1 expression intensity showed strong staining consistent with the control groups, and the percentage of positive cells was determined as 11.52% and 12.49%, respectively. When the data were evaluated statistically, it was observed that there was a significant difference between the groups (*p* < 0.001). The D, D+2D-CM, and D+3D-CM groups showed significantly lower Nkx6.1-positive cell percentage in the pancreatic islets than the C, C+2D-CM, and C+3D-CM groups. The positive cell percentage tended to increase in the D+2D-CM and D+3D-CM treatment groups, but this increase was not at a level that would make a significant difference (Fig. 6B). In addition, when the exocrine pancreas was examined in the sections, it was noted that there were Nkx6.1-positive cells among the acinar cells in the D+2D-CM and D+3D-CM groups (Fig. 7) importantly.

#### Pdx1

The Pdx1 marker, which plays a critical role in pancreatic development, endocrine cell differentiation, and maintenance of mature β-cell function, was labeled immunohistochemically (Fig. 6A) to evaluate the effects of conditioned medium on the protection and regeneration of β-cells. Then, five sections for each animal were analyzed, and the average percentage of Pdx1-positive cells in an islet was calculated (Fig. 6C). Accordingly, Pdx1-positive cells were found in 76.99% in the C group, 77.54% in the C+2D-CM group, and 77.54% in the C+3D-CM group. It was observed that this positivity decreased to 17.11% in the D group, while the percentage of Pdx1-positive cells in the D+2B-CM and D+3B-CM treatment groups increased to 29.72% and 32.20%, respectively. As a result of the statistical evaluation, it was observed that there was a significant difference between the groups (*p* < 0.001). The D, D+2D-CM, and D+3D-CM groups showed significantly lower Pdx1-positive cell percentage in the pancreatic islets than the C, C+2D-CM, and C+3D-CM groups. Furthermore, it was found that the percentage of Pdx1-positive cells in the D+2D-CM and D+3D-CM treatment groups increased compared to the D group, and this increase was statistically significant.

#### Insulin

For the analysis of insulin-producing β-cells in the islets, the pancreatic sections were labeled with the anti-insulin antibody (Fig. 6A). An average of 7 sections for each animal was examined during the analysis. The percentage of the insulin-positive area in an islet within the total islet area was calculated with the ImageJ program, and the results were compared (Fig. 6D). Accordingly, 74.82% area in the C group’s islets, 74.90% in the C+2D-CM group’s islets, and 79.60% in the C+3D-CM group’s islets were insulin-positive. It was observed that this positivity decreased to 16.63% in the D group, while the percentage of insulin-positive areas in the D+2D-CM and D+3D-CM treatment groups increased to 20.54% and 30.22%, respectively. As a result of the statistical evaluation, it was observed that there was a significant difference between the groups (*p* < 0.001). The D, D+2D-CM, and D+3D-CM groups showed a significant decrease in the percentage of the insulin-positive area of islets compared to the C groups (C, C+2D-CM, and C+3D-CM). In addition, the increase in insulin positivity in the D+3D group was statistically significant. Moreover, the exocrine pancreas parts were examined in the sections. It was discovered that there were cells showing insulin positivity between the acinar cells in the D+2D-CM and D+3D-CM groups (Fig. 7).

## Discussion

Here, we demonstrate for the first time the therapeutic effects of a conditioned medium prepared by culturing human umbilical cord-derived MSCs in 2D and 3D environments on immunomodulation and β-cell protection/regeneration in an in vivo T1D model. Mesenchymal stem cells (MSCs) have been prominent cells with their differentiation abilities since they were discovered. However, the factors secreted by MSCs have been revealed recently. Due to the secretomes, it is now emphasized that MSCs exert their therapeutic effects through paracrine mechanisms [7–9], and the studies need to be further advance. As it is known, MSCs have anti-apoptotic, anti-inflammatory, and cytoprotective effects and play a role in immunomodulatory and immunosuppressor mechanisms [26]. Considering these, MSCs as crucial cells, and their CMs appear to respond to the treatment needs of T1D with their characteristic features.

First, the isolation and characterization of MSCs required for preparing conditioned media to be used therapeutically in the T1D model were performed in this study. MSCs used during conditioned medium preparation were isolated from the umbilical cord of a single donor. Then, the isolated cells were proven to be MSCs by performing the characterization experiments. Donor-dependent differences in isolated cells could constitute a problem for adult tissues such as bone marrow and adipose tissue. Many factors affect the biological characteristics of cells in adult tissues, especially age. However, since the umbilical cord is a primitive tissue, individual differences are minimized. Raileanu et al. investigated the donor-dependent difference in umbilical cord mesenchymal stem cells in a large-scale study (*n* = 71) and found consistent results. The results showed that MSCs from different donors all have typical MSC properties, similar cytokine profiles, and good therapeutic potential [27]. In our previous studies, we examined the MSC characteristics, which we isolated from different donors, for eight passages, and we did not detect any individual differences [6, 18]. For the characterization of these cells, the following criteria are expected to be met since there is no single marker to prove that the isolated cells are MSCs [28]. MSCs should be plastic-adherent under standard culture conditions and differentiate into trilineage mesenchymal differentiation pathways, and the cells must have a specific cell surface antigen expression. After the cells were shown to be plastic adherent, they were characterized by immunophenotypic markers. The positive and negative markers for immunophenotypic evaluation were chosen based on the referenced studies. The studies in the literature showed that, generally, a set of antigens among these antigens are used for characterization [8, 29–31]. Nagamura-Inoue et al. described the biomarkers of umbilical cord MSCs [32], and the selected markers are compatible with the markers specified in this study. As a result of these experiments, CD44 and CD90 positivity over 95% of the cells and CD34 and CD45 negativity below 5% were other parameters indicating that the isolated cells were MSCs. Finally, we demonstrated the isolated cells’ adipogenic and osteogenic differentiation abilities. Thus, by choosing two of the three primary tissues of mesoderm origin, we showed that our cells have MSC characteristics, in line with the studies in the literature [29, 33–35].

Following the isolation and characterization stages, 2D-CM and 3D-CM were obtained as a result of culturing the same number of cells simultaneously, and when protein concentrations were compared, it was determined that there was a 34% increase in 3D-CM compared to 2D-CM. The data in the literature show that MSCs cultured on scaffolds secrete a higher rate of secretome [8, 16, 36], consistent with this finding. Because the 3D environment provides a more suitable environment for the normal physiological conditions of the cells, the biological behaviors of the cells such as phenotype, proliferation, migration, and protein expression can be regulated more effectively in 3D environments. The regulation could lead to a change in the amounts of paracrine factors and paracrine profile [8, 20, 37]. IL-4, IL-10, IL-17, and IFN-γ, which are closely related to T1D pathogenesis, were selected for analysis in the serum of rats. IL-4 level was found to be 17.11 pg/ml in 2D-CM and 23.25 pg/ml in 3D-CM. However, IL-10, IL-17, and IFN-γ could not be detected at a measurable level in CMs. While these analyses were limited beneficent for us to detect conditioned medium content, they were necessary to understand whether a possible cytokine increase seen in in vivo experiments was due to the applied conditioned medium. In addition, these findings are valuable for discussions about whether MSCs are capable of secreting cytokines, which is still controversial in the literature. The cytokine secretion profiles of MSCs derived from different sources and cultured under different conditions still contain contradictions. Some studies have reported that MSCs do not secrete cytokines such as IL-4 and IL-10 under normal conditions [38] and can detect only inflammatory conditions and the presence of cytokines [39]. However, studies showing that amniotic fluid and adipose tissue-derived MSCs secrete cytokines such as IL-4, IL-6, and IL-10 are also available in the literature [40, 41]. In this context, CMs analyzed showed that hUC-derived MSCs secrete IL-4, but it is evident that future studies need to examine the relevant cytokines of CM contents prepared under different circumstances from umbilical cord MSCs.

The in vivo immunomodulatory effects of CMs obtained from MSCs were evaluated by Treg population analysis in T-cells isolated from the spleen and pro-inflammatory and anti-inflammatory cytokine analysis in the peripheral blood. According to these analyses, it was observed that the Treg population decreased in group D, and the Th1-related IFN-λ and Th17-related IL-17 cytokines increased in the peripheral blood. This situation develops due to the chronic inflammation state and indicates that the fine balance between autoreactive T-cells and regulatory T-cells is disturbed because of the T1D model [42]. The increase in both cytokines with the induction of diabetes may have occurred as a result of the inability to inhibit Th1 and Th17 due to the decrease in Tregs [43]. In order to revert the imbalance mechanism of T1D, it may need to be restored. In the evaluations made after applying 2D-CM and 3D-CM for 4 weeks to the diabetic groups, it was determined that there was a significant increase in the Treg population. In addition, a significant increase in Treg cells was observed in the C+3D-CM group. These results imply that MSC-CMs could induce Treg cells through the molecules they secrete and that was critical in restoring the impaired T-cell mechanism and the balance between autoreactive T-cells and Tregs. As for the results of cytokine analysis, it was determined that there was an increase in IFN-λ and IL-17 levels in the treatment groups (D+2D-CM, D+3D-CM), and the increase in IFN-λ was statistically significant. Contrary to the general paradigm, the increase in these pro-inflammatory cytokines, known to play essential roles in diabetes pathogenesis, was remarkable after the treatment [44]. Since it was shown that IL-17 and IFN-λ were not found in the CMs administered to animals, it can be said that the cytokine increases were not due to the direct effects of the applied treatment but the result of the immune cells’ regulating abilities. T1D has traditionally been considered a Th1-mediated pathology in which Th2 cells play a protective role. However, studies in the literature show that both Th1 and Th2 responses have inducing and protective effects on the pathogenesis of T1D [45]. It has been demonstrated that a deficiency in IFN-λ or its receptor did not prevent the development of diabetes, only a slight delay [46]. Also, some studies showed that the injection of recombinant IFN-λ did not accelerate diabetes and could even restore normoglycemia [47, 48]. Moreover, the literature review indicates that diabetogenic T-cells and IFN-λ can paradoxically help islet-specific Tregs provide sustained protection from diabetes [49, 50]. In another study, when mycobacterial adjuvant treatment was applied to non-obese diabetic (NOD) mice, it was reported that IFN-λ and IL-17 levels increased with the protection from the disease [51]. It becomes clear that the traditional Th1 mechanism should be questioned, because different data and approaches in the literature state that it is difficult to name a cytokine as pro-inflammatory or anti-inflammatory because all cytokines act at different stages of both mechanisms [39]. Understanding cytokines, the cells with which they interact, and the disease mechanisms with which they are associated will only be possible with the cumulative effect of new studies that will be included in the literature. When the levels of IL-4 and IL-10 in the peripheral blood were examined, it was observed that Treg-related IL-10 levels could not be detected in the C groups and increased significantly in the D+2D-CM group. In the D+3D-CM group, despite the increase in the Treg cell population in the spleen, IL-10 was not detected in the peripheral blood. The situation may be related to the Treg cells, which increase in number in the spleen; have not been transferred to the peripheral blood at the same rate; or their activation has not been completed even if the transition has taken place. It also brings to mind other possibilities related to unidentified CM’s content except for the Treg cell rates. In addition, cytokine levels in the peripheral blood interact with many factors and show many changes. Although the IL-10 level was increased in the D+2D-CM group, its absence in the D+3D-CM group may have developed due to the changes in cytokines and growth factors in the 3D CM content. Considering the diversity of cytokines and growth factors in CMs, revealing the content profile in future studies will make it possible to explain possible interactions. Finally, it was observed that the IL-4 level was not detectable in the C groups but increased relatively in the C+2D-CM, C+3D-CM, and D groups, while it increased significantly in the D+2D-CM and D+3D-CM treatment groups. It was suggested that the release of IL-4 from Th2 cells induced due to the increased Treg population helps immunomodulation [2]. Considering all these findings, it can be suggested that 2D-CMs and 3D-CMs improve the autoimmune T1D model by regulating the imbalance between Th1, Th2, T17, and Treg cells.

In order to evaluate the ability of MSC-CM to induce β-cell protection and regeneration by triggering endogenous mechanisms, two major transcription factors, Pdx1 and Nkx6.1, were examined immunohistochemically in the pancreatic sections. Accordingly, the rate of positive cells detected between 74 and 78% in the control groups for both antibodies is consistent with the information that the rate of β-cells in rodents is 70–80% [52]. When the results of the Nkx6.1 were examined, it was seen that the percentage of positive cells in the D group showed a significant decrease. The rate tended to increase in the treatment groups, but this increase was not statistically significant. In the results of the Pdx1 marker, the percentage of positive cells in the D group showed a significant decrease compared to the C groups. In the meantime, the D+2D-CM and D+3D-CM treatment groups increased significantly compared to the D group. When these two markers are evaluated together, it can be said that the applied CMs are effective in β-cell homeostasis and regeneration. Following these findings, the insulin level of β-cells in the islets was evaluated immunohistochemically. Accordingly, the percentage of insulin-positive areas in the control groups was consistent with the percentage of positive cells in Pdx1 and Nkx6.1 markers. The percentage of insulin-positive areas in the D group decreased significantly compared to the C groups, which increased in the treatment groups. It was determined that this increase was significant in the D+3D-CM group compared to the D and D+2D-CM groups. Based on these results, it can be said that MSC’s secretome could help β-cell protection and induce β-cell regeneration by playing a role in immunomodulation and also with their anti-inflammatory, anti-apoptotic, and cytoprotective properties. A study examining the anti-diabetic effects of MSCs reported that the islet structures and β-cells of diabetic mice treated with MSCs improved, but no MSC was found in the pancreas. On the contrary, it has been said that MSCs localize to secondary lymphoid organs such as the spleen and lymph nodes, thus contributing to recovery by reversing the disrupted T-cell mechanism [53]. In addition, many studies have shown that MSCs have no or minimal ability to transdifferentiate into β-cells [53, 54]. In this context, our findings showed that MSCs could heal β-cells only with their secretomes and trigger new β-cell formation in the exocrine pancreatic regions, and it has brought a new insight to the literature on the anti-diabetic effects of MSCs. It is known that the increase in β-cells in the treatment groups may have been caused by the mechanisms such as β-cell proliferation and regeneration, α-cell transdifferentiation, or neogenesis of the progenitor cells [55, 56]. Moreover, Nkx6.1-positive and insulin-positive cells in the exocrine pancreas in D+2D-CM and D+3D-CM groups suggest that CMs may have triggered local progenitor cell niches. In addition, since the pancreatic exocrine cells and β-cells both develop from the foregut endoderm, which means they have the same embryological origin [57], it is thought that these newly formed β-cells may have arisen by reprogramming the exocrine cells into insulin-expressing β-cells [56]. The available findings are insufficient to comment on this issue, but it draws attention as an important finding that needs to be examined with further research.

## Conclusion

In this study, it has been shown that human umbilical cord-derived MSC-CM induces the Treg cell population regulates cytokine release and increases the number of Pdx1, Nkx6.1, and insulin-positive cells in the pancreatic islets in the T1D model. These data revealed that a conditioned medium could exert dual therapeutic effects on the T1D model in the context of immunomodulation and β-cell protection and regeneration. Changes in the islet structure, expression levels of β-cell markers in CM-treated groups, and the localization of positive cells in the pancreatic tissue have provided new approaches to β-cell homeostasis and regeneration. In addition, the increase in the Treg population and the number of Pdx1, Nkx6.1, and insulin-positive cells in the islets were higher in the 3D-CM group than in the 2D-CM group. This finding together with higher protein content suggested that the therapeutic effects of the conditioned medium could be enhanced by growing MSCs on 3D scaffolds with a specific topography.

## Data Availability

The datasets used and/or analyzed during the current study are available from the corresponding author upon reasonable request.

## Abbreviations

2D: 2-Dimensional
3D: 3-Dimensional
2D-CM: 2-Dimensional conditioned medium
3D-CM: 3-Dimensional conditioned medium
APC: Allophycocyanin
BCA: Bicinchoninic acid
C: Control group
C+2D-CM: Control + 2-dimensional conditioned medium group
C+3D-CM: Control + 3-dimensional conditioned medium group
CD: Cluster of differentiation
CO_2_: Carbon dioxide
D: Diabetes group
D+2D-CM: Diabetes + 2-dimensional conditioned medium group
D+3D-CM: Diabetes + 3-dimensional conditioned medium group
EDTA: Ethylenediaminetetraacetic acid
ELISA: The enzyme-linked immunosorbent assay
FACS: Fluorescence-activated cell sorting
FBS: Fetal bovine serum
FITC: Fluorescein isothiocyanate
FoxP3: Forkhead box P3
HGF: Hepatocyte growth factor
HLA-G: Human leukocyte antigen G
H2O2: Hydrogen peroxide
H&E: Hematoxylin and eosin
IFN-γ: Interferon gamma
IL: Interleukin
MSC: Mesenchymal stem cell
NK: Natural killer
Nkx6.1: NK6 homeobox 1
NOD: Non-obese diabetic
PBS: Phosphate-buffered saline
Pdx1: Pancreatic and duodenal homeobox 1
PE: Phycoerythrin
PGE2: Prostaglandin E2
RPMI: Roswell Park Memorial Institute
STZ: Streptozotocin
T1D: Type 1 diabetes
TBS: TRIS-buffered saline
TC: Cytotoxic T-cell
TH: T helper cell
TGF-β: Transforming growth factor beta
Treg: Regulatory T-cells
VEGF: Vascular endothelial growth factor
